# Assessing quality of life in people with HIV in Spain: psychometric testing of the Spanish version of WHOQOL-HIV-BREF

**DOI:** 10.1186/s12955-019-1208-8

**Published:** 2019-08-19

**Authors:** Maria Jose Fuster-RuizdeApodaca, Ana Laguía, Kelly Safreed-Harmon, Jeffrey V. Lazarus, Santiago Cenoz, Julia del Amo

**Affiliations:** 1 Spanish Interdisciplinary AIDS Society (Sociedad Española Interdisciplinaria del Sida SEISIDA), C/ Doctor Fleming 3, 28036 Madrid, Spain; 2 Facultad de Psicología, Universidad Nacional de Educación a Distancia (UNED), Calle Juan del Rosal, 10, ES-28040 Madrid, Spain; 3 Barcelona Institute for Global Health (ISGlobal), Hospital Clínic, University of Barcelona, Barcelona, Spain; 4 ViiV Healthcare, Madrid, Spain; 5 Centro Nacional de Epidemiología, Instituto de Salud Carlos III, Madrid, Spain; 6 Plan Nacional del Sida y otras ITS, Dirección General de Salud Pública, Calidad e Innovación, Ministerio de Sanidad, Consumo y Bienestar Social, Madrid, Spain

**Keywords:** HIV, Health-related quality of life, WHOQOL-HIV-BREF

## Abstract

**Background:**

The assessment of health-related quality of life (HRQoL) in people living with HIV (PLHIV) has become crucial to evidence-based practice. The goals of this study are to analyze the psychometric properties and evidence of the validity of the Spanish version of WHOQOL-HIV-BREF in a sample of PLHIV in Spain and to examine the more impaired HRQoL facets and dimensions and identify the PLHIV who show the most vulnerable profile.

**Methods:**

A total of 1462 PLHIV participated in an observational cross-sectional ex-post-facto study. Data were collected at 33 Spanish sites through an online survey. In addition to measuring HRQoL, the study used other tools to measure treatment adherence (CEAT-VIH 2.0 version), psychological well-being (GHQ-12) and HIV-related stigma (HSSS). Cronbach’s alpha, first- and second-order confirmatory factor analysis (CFA), the Pearson coefficient and one-way ANOVA were used to evaluate reliability, construct validity and concurrent and known-group validity, respectively. Differences according to the socio-demographic and epidemiological profiles of participants were analyzed.

**Results:**

First- and second-order CFAs confirmed a six-domain first-order structure of the Spanish version of WHOQOL-HIV-BREF and one second-order factor related to overall HRQoL with an acceptable fit to the data, although some minor changes would improve it. The six-domain structure showed an acceptable internal consistency (Cronbach’s alpha ranged from .61 to .81). Significant moderate to large correlations between domains and overall HRQoL, adherence, psychological well-being and negative self-image were found. Significant differences were found according to participants’ self-reported CD4+ cell count in several HRQoL facets and domains. Being female, heterosexual, having low socio-economic and educational statuses, having acquired HIV through an unsafe injection and living more years with HIV were related to poorer HRQoL. PLHIV older than 50 presented lower scores in 19 HRQoL facets.

**Conclusions:**

This study demonstrates that the Spanish version of the WHOQOL-HIV-BREF is a valid instrument. It also presents the most recent data about HRQoL in PLHIV in Spain with the largest sample to date.

**Electronic supplementary material:**

The online version of this article (10.1186/s12955-019-1208-8) contains supplementary material, which is available to authorized users.

## Background

An estimated 36.7 million people are living with HIV worldwide [1]. Spain has an estimated 145,000 people living with HIV (PLHIV) [2], and there were 3353 new HIV diagnoses in 2016 [3]. While AIDS cases have declined in Spain in recent years with the use of effective antiretroviral therapy (ART) regimens, late diagnosis remains a problem. In more than one-quarter of new diagnoses in 2016, the person diagnosed had a CD4 cell count of less than 200, indicating advanced disease [3].

Improvements in ART have resulted in increased life expectancy for many PLHIV. Nonetheless, HIV infection and its related problems still have a notable impact on health-related quality of life (HRQoL), even in people who are virally suppressed as a result of taking ART [4]. Helping PLHIV achieve good outcomes in regard to their HRQoL requires understanding its determinants in this population. Studies have identified a number of factors that are consistently associated with HRQoL among PLHIV, including ageing, immunological status, the presence of symptoms, treatment adherence, depression, social support, employment and HIV-related stigma [5, 6].

In this context, the precise assessment of HRQoL with a valid measure has become crucial to the improvement of quality of life of PLHIV [7]. Additionally, HRQoL assessment has a major role in evaluating intervention outcomes [8]. Several instruments, both generic and HIV-specific, have been used to measure HRQoL in PLHIV. Generic instruments such as the widely used EQ-5D and SF-36 have the advantage of yielding findings that can be compared to HRQoL findings for the general population. However, HIV-specific instruments have shown greater sensitivity than generic ones [9]. WHOQOL-HIV-BREF [8] is considered to be one of the most promising of the HIV-specific instruments because of its psychometric properties, relevance to PLHIV and cross-cultural validity [9].

WHOQOL-HIV-BREF is the short version of WHOQOL-HIV [7]. Both instruments contain facets (individual components) of the generic WHOQOL measure [10] as well as HIV-specific facets. WHOQOL-HIV-BREF includes 29 items covering six domains: physical, psychological, level of independence, social, environmental and spiritual. There is also a two-item general facet.

The generic WHOQOL instrument has been validated in Spain [11]. However, there are no studies reporting the use of WHOQOL-HIV-BREF with Spanish PLHIV. This study aimed to assess the psychometric properties and evidence of the validity of the Spanish version of WHOQOL-HIV-BREF in a large sample of people with HIV in Spain. Furthermore, it aimed to determine which HRQoL facets and dimensions are most impaired and which groups of PLHIV are more vulnerable to these outcomes.

## Methods

### Study population and procedures

An observational cross-sectional ex-post-facto study was conducted in which 1462 PLHIV participated. They were recruited by convenience sampling. The general inclusion criteria were positive HIV diagnosis, being at least 18 years old, on antiretroviral therapy (ART) for at least one year, and not having any severe psychiatric or cognitive disorder. Data were collected between October 2016 and April 2017.

An online survey was designed using the Qualtrics survey platform (available at: www.qualtrics.com). Qualtrics is a private online survey development platform that allows the creation of surveys which can be accessed through a link. In the present study, our survey was self-administered with the support of tablet computers. Service providers from 33 service delivery points across Spain (hospitals and NGOs) collaborated in the participants’ recruitment and data collection. During their medical consultations or when attending various services, the collaborating service providers explained the goals of the study to the participants, requesting their participation and obtaining their informed consent. The rate of refusal to participate in the study varied across centers, ranging from 0 to 18%, with an average around 7%. The main reasons argued for the refusal were not having enough time, the survey length or lack of skills to use tablets. Participants were compensated with 15 euros.

The Ethics Committee of the Hospital Clínico of Valencia approved the research protocol in March 2016. All study procedures were conducted in accordance with the 1964 Helsinki Declaration (revised in 1996) [12].

### Measures

#### WHOQOL-HIV-BREF

WHOQOL-HIV-BREF has 31 items covering six domains: physical health; psychological health; level of independence; social relationship; environmental health; and spirituality, religion and personal beliefs (SRPB) [8]. Responses to all items are given on a 5-point scale. Items that ask about negative perceptions and experiences, such as “How much do you fear the future?” are reverse-coded for scoring. Thus, higher scores for all items indicate better quality of life. The average score for each domain is multiplied by four, yielding domain scores that range from 4 to 20 [13].

Several studies have examined the validity and psychometric properties of WHOQOL-HIV-BREF in different languages and countries. (A summary of these studies is presented in Additional file 1). They have found the instrument to have good psychometric properties and have also found evidence of its validity. The Spiritual, Religion and Personal Beliefs domain (SRPB) is the one that exhibited the lowest reliability (under .70) and discriminative power in most of the studies [8, 14–18], although it is the domain which contains more HIV-specific items measuring existential concerns relating to HIV.

Because WHOQOL-BREF has been validated in a Spanish study population [19], only the translation of the HIV-specific items was needed (see Additional file 2). The HIV-specific items collect information about the bother caused by physical problems related to HIV infection, HIV-related stigma, and fears related to the future and to death (“How much are you bothered by any physical problems related to your HIV infection?,” “To what extent are you bothered by people blaming you for your HIV status?,” “How much do you fear the future?,” “How much do you worry about death?,” and “To what extent do you feel accepted by the people you know?”). These items were extracted from five HIV facets of the WHOQOL-HIV long form and then integrated with the WHOQOL-BREF to complete the 31-item WHOQOL-HIV BREF [8]. They were translated following the criteria of the International Test Commission [20]. A backward translation was performed by two expert translators. In addition, a person with HIV reviewed the translation.

#### Questionnaire to evaluate the adherence to HIV therapy (CEAT-VIH 2.0 version)

The validated Spanish version of the Questionnaire to Evaluate the Adherence to HIV Therapy (CEAT-VIH 2.0 version) [21–23] was used. This scale is comprised of 17 items rated on a 5-point scale. Negative items were reverse-coded. A composite of all items (total score) was calculated, with higher the scores indicating higher treatment adherence. A systematic review of the psychometric properties of the CEAT-VIH including 20 studies revealed an adequate internal consistency as well as no floor or ceiling effects [23]. Additionally, evidence of validity comprised criterion-related validity (e.g., HIV viral load, length of time with continuous undetectable HIV viral load, days of missed doses, number of pills per day, and adherence assessed by the pharmacist or physician); responsiveness, sensitivity, and specificity; and patterns of convergence and divergence (e.g., negative mood, depression, anxiety and stress were negatively associated with CEAT-VIH scores whereas positive correlations with CEAT-VIH scores were found for perceived social support and quality of life outcomes).

#### General Health Questionnaire (GHQ-12)

The validated Spanish version of the General Health Questionnaire (GHQ-12) was used [24]. Items are rated on a 4-point scale, with higher scores indicating better psychological health. In previous studies, this scale has shown adequate reliability and validity in the Spanish general population [24].

#### HIV-related stigma

Five items of the Negative Self-image dimension and three items of the Disclosure Concerns dimension of the Spanish HIV Stigma Scale (HSSS) were used [25]. These items were selected for having higher validity constructs in the validation study of the scale. A previous study in Spain revealed that this scale shows good internal consistency and good construct validity, including content and criterion validity [25]. The items are rated on a 4-point scale.

Self-reported questions related to health status such years living with HIV, lymphocyte CD4 count and viral load copies were included in the survey. Moreover, the questionnaire also measured socio-demographic information.

### Data analysis

Completing most items of the online questionnaire in the Qualtrics survey platform was programmed to be compulsory. Only items related to some sensitive characteristics of the participants were allowed to be skipped. Thus, there were no missing values in the tools used to measure the variables under analysis.

To test the construct validity, first-order confirmatory factor analysis (CFA) was used to assess the fit of the Spanish version of the WHOQOL-HIV-BREF to the six-dimension original structure (Model 1, [8]). Next, second-order CFA was performed to determine whether the six first-order factors could be explained by a higher-order latent factor associated with HRQoL. Previous studies showed that one of the items of the SRPB dimension presented a low facet-domain correlation [18] or factor loading [14, 17] and was saturated in the psychological domain rather than in the existential domain when exploratory factor analysis (EFA) was conducted [16]. Thus, we tested an alternative model (Model 2) allowing one of the items of the SRPB dimension—feelings of personal meaning—to load in the Psychological Health latent dimension. The robust unweighted least square method was used because the items in the scale did not meet the assumption of normality. Goodness of fit was evaluated using the goodness of fit index (GFI), the adjusted goodness of fit index (AGFI), the comparative fit index (CFI)*,* the standardized root mean square residual (SRMR) and the standardized root mean square error of approximation (RMSEA). Also, the consistent Akaike information criterion (CAIC) was used to compare the alternative models*.* According to Hu and Bentler [26], the models are considered to have a good fit when the goodness of fit indexes (GFI and AGFI) and CFI > .90, RMSEA < .08 and SRMR <.08. The reliability of each domain was assessed using Cronbach’s α coefficient.

Pearson’s correlation analysis between domain scores and the general health dimension of WHOQOL-HIV-BREF was performed for convergent validity. To assess concurrent validity, we examine the association between domain scores and the criterion variables measured. We expected to find positive correlations between domain scores and CEAT-VIH and GHQ-12. We also expected to find negative correlations between domain scores and HIV-related stigma dimensions.

Known-group validity was used to assess the capacity of WHOQOL-HIV-BREF to discriminate among subgroups of participants according to their immunological (CD4 count) and virological (viral load copies) status. It was expected that participants with higher CD4 count and undetectable viral load would have higher HRQoL domain scores.

Finally, differences according to the socio-demographic and epidemiological profiles of study participants were analyzed. For the sake of simplicity and clarity, only differences in the domains were assessed in most of the characteristics of the participants, and only *p*-values are shown. However, differences in all HRQoL facets were tested according to age and sex. It was done because of the current relevance of analyzing HRQoL in aging PLHIV and the UNAIDS recommendations about disaggregation of data according to relevant socio-demographic characteristics [27]. To test differences by age, a cut-off point of 49 years (≤ 49 vs. ≥ 50 years) was established. T-test and one-way variance analysis were used for these analyses.

Regarding the data analysis software, LISREL (LInear Structural RELations) 8.7 program and its companion preprocessor program PRELIS for Windows were used for the CFAs. LISREL is an application for structural equation modeling developed by K. G. Jöreskog and D. Sörborm [28]. PRELIS is an application for data manipulation, data transformation, data generation, computing moment matrices and imputation by matching. A widely used program for statistical analysis in social science, IBM SPSS Statistics 22 [29], was selected for the remaining analyses.

## Results

### Sample characteristics

Socio-demographic and health data of the participants are shown in Table 1. The epidemiological profile found was concordant with Spanish epidemiological data for PLHIV [3].

**Table 1:** Sociodemographic and clinical characteristics of the participants (*N* = 1462)

Sociodemographic and clinical variables	%
Gender	
Males	79.3
Females	19.3
Transsexual	1.4
Age, years, mean (*M* ± *SD*)	45.0 ± 10.2
Education level	
No studies	3.9
Elementary School	26.2
High School	33.1
University degree	30
Other	6.8
Work situation	
Working (full time)	33.9
Working (part-time)	10.8
Unemployed	18.2
Retired/impaired	16.4
Other	20.7
Monthly incomes	
None	13.3
< 1000 €	37.7
1001 €–1500 €	29.3
1501 €–2000 €	7.5
> 2000 €	12.2
Sexual behavior	
Heterosexual	39.7
Homosexual	52.8
Bisexual	4.2
Others	1.9
No answer	1.3
Transmission route	
Sexual intercourse	68.2
Sharing injection materials	18.6
Unknown (various concomitant practices)	10.2
Other	3
CD4 cell count, cells/mm^3^	
< 200	4.8
201–400	10.5
> 400	68.1
Unknown	16.6
Duration of infection, years, mean (*M* ± *SD*)	13.8 ± 9.6
Years taking antiretroviral therapy (*M* ± *SD*)	11.3 ± 8.3
Undetectable plasma viral load	90.4

Note: Data in percentages unless otherwise stated

### WHOQOL-HIV-BREF scores

The descriptive statistics of each item and domain are displayed in Table 2. The mean scores of the WHOQOL-HIV-BREF domains were calculated according to the WHOQOL-HIV Instrument Users Manual [30].

**Table 2:** Descriptive statistics and reliability of the domains and items (*N* = 1462)

Domains and items	Mean (± *SD*)	Skewness	Kurtosis	Corrected item-domain correlation	Cronbach’s α if item is deleted
Overall QoL/General Health (α = .77)	14.9 ± 3.6				
How would you rate your QoL?	3.6 ± 1.06	−.40	−.63	.63	
How satisfied are you with your health?	3.8 ± .93	−.94	.78	.63	
Physical health (α = .73)	15.5 ± 3.2				
Pain and discomfort^a^	3.9 ± 1.1	−.87	−.39	.58	.64
Symptoms of HIV^a^	4.2 ± 1.0	−1.2	.55	.54	.66
Energy and fatigue	3.9 ± .97	−.77	.05	.56	.65
Sleep and rest	3.3 ± 1.1	−.38	−.79	.43	.73
Psychological health (α = .81)	14.9 ± 3.0				
Positive feelings	3.9 ± 1.0	−.92	.29	.66	.75
Concentration ability	3.5 ± .96	−.52	−.40	.46	.81
Bodily image self-acceptance	3.9 ± .96	−.91	.49	.52	.79
Self-satisfaction	3.8 ± 1.0	−.82	.16	.73	.73
Negative feelings^a^	3.3 ± 1.0	−.11	−.71	.61	.77
Level of Independence (α = .67)	15.5 ± 3.2				
Dependence on medication^a^	3.4 ± 1.5	−.39	−1.3	.29	.79
Mobility	4.4 ± .81	− 1.6	2.9	.46	.62
Activities of daily living	3.9 ± .96	−.81	.25	.64	.51
Work capacity	3.7 ± 1.1	−.83	−.01	.60	.51
Social relations (α = .75)	15.0 ± 3.2				
Social inclusion	4.1 ± .91	−1.1	1.3	.51	.71
Personal relationships	3.7 ± 1.0	−.73	−.03	.63	.64
Sexual satisfaction	3.2 ± 1.2	−.37	−.95	.46	.75
Social support	3.9 ± 1.0	−.96	.38	.61	.65
Environmental health (α = .81)	15.3 ± 2.5				
Physical safety and security	3.7 ± .96	−.64	−.05	.54	.79
Physical environment	3.9 ± .90	−.93	.87	.57	.79
Financial resources	3.0 ± 1.0	−.24	−.64	.51	.79
Information for daily living	4.1 ± .84	−.90	.93	.56	.79
Participation in leisure activities	3.7 ± 1.0	−.68	−.23	.61	.78
Home environment	3.9 ± 1.0	−1.0	.82	.62	.78
Accessibility of health services	4.2 ± .80	− 1.0	1.1	.46	.80
Transport	3.8 ± 1.0	−.83	.29	.38	.81
Spirituality/Personal beliefs (α = .61)	14.5 ± 3.5				
Personal life meaning	4.0 ± 1.0	−1.0	.49	.24	.63
Forgiveness and blame^a^	3.3 ± 1.6	−.33	−1.4	.32	.62
Concerns about the future^a^	3.4 ± 1.2	−.36	−.88	.57	.40
Death and dying^a^	3.7 ± 1.2	−.66	−.57	.47	.48

^a^ Reversed items recoded

The skewness and kurtosis coefficients of most items ranged from − 1.00 to 1.00. Some of them showed somewhat higher coefficients, but coefficients of less than 1.5 can also be considered adequate [31]. The item showing highest kurtosis (2.9) was the one measuring mobility.

The facets showing the lowest scores were *financial resources, sexual satisfaction, sleep and rest, negative feelings,* and *forgiveness and blame*. Across domains, Physical Health and Level of Independence showed the highest scores while the SRPB domain presented the lowest score.

### Validity of the Spanish version of the WHOQOL-HIV-BREF

#### Construct validity

The results of the first-order CFA confirmed the six-domain factor structure with an acceptable fit to the data (Table 3). The results of the second-order CFA confirmed the six first-order dimensions and one second-order factor related to overall HRQoL, also with acceptable model fit statistics. Most of the standardized loadings were higher than 0.5, the level considered adequate [32]. However, lower loadings were found in the facet *dependence on medication* in the Level of Independence domain; the facet *transport* in the Environmental Health domain; and the facets *forgiveness and blame* and *death and dying* in the Spirituality.

**Table 3:** Standardized estimations for the six-domain first-order and HRQoL second-order structure Confirmatory Factor Analysis (CFA) model

	First Order (λ)		Second Order (γ)	
Domains and items	Model 1	Model 2	Model 1	Model 2
Physical health			.92	.93
Pain and discomfort^a^	.62	.63		
Symptoms of HIV^a^	.60	.60		
Energy and fatigue	.88	.88		
Sleep and rest	.65	.65		
Psychological health			.97	.94
Positive feelings	.84	.85		
Concentration ability	.57	.57		
Bodily image self-acceptance	.67	.68		
Self-satisfaction	.86	.87		
Negative feelings^a^	.69	.70		
Religion, spirituality and personal beliefs (personal life meaning)	–	.81		
Level of Independence			.94	.94
Dependence on medication^a^	.38	.39		
Mobility	.73	.73		
Activities of daily living	.87	.87		
Work capacity	.81	.81		
Social relations			.91	.91
Social inclusion	.70	.70		
Personal relationships	.81	.81		
Sexual satisfaction	.65	.65		
Social support	.71	.71		
Environmental health			.92	.92
Physical safety and security	.82	.81		
Physical environment	.69	.69		
Financial resources	.56	.56		
Information for daily living	.69	.69		
Participation in leisure activities	.74	.74		
Home environment	.71	.71		
Accessibility of health services	.51	.51		
Transport	.40	.40		
Spirituality/Personal beliefs			.86	.50
Religion, spirituality and personal beliefs (personal life meaning)	.89	–		
Forgiveness and blame^a^	.36	.56		
Concerns about the future^a^	.51	.87		
Death and dying^a^	.35	.60		
*SB-χ2 (Satorra-Bentler Chi-square)*	3210.71	2691.78	3435.87	2906.40
*Degrees of freedom*	362	362	370	370
*p*	< .001	< .001	< .001	< .001
*RMSEA* (IC 90%)	0.073 (0.071; 0.076)	0.066 (0.064; 0.069)	0.075 (0.073; 0.078)	0.068 (0.066; 0.071)
*SRMR*	0.064	0.058	0.068	0.062
*GFI*	0.98	0.98	0.98	0.98
*AGFI*	0.97	0.98	0.97	0.98
*CFI*	0.97	0.97	0.97	0.97
*NFI*	0.97	0.97	0.97	0.97
*CAIC*	3815.70	3296.77	3974.56	3445.10

Notes: *N* = 1462. Estimation of the robust unweighted least squares. SB-χ^2^: Satorra-Bentler Chi-square. *df*: degrees of freedom. Model 1: dimensions according to the original scale. Model 2: allowing the item *religion, spirituality and personal beliefs* (feeling that life is meaningful) to load in the Psychological Health domain instead of the SRPB domain

^a^ Reversed items recoded

All factor loadings *p* < .05

Results of the alternative model tested (Model 2) showed a high standardized loading for the item *religion, spirituality and personal beliefs* in the Psychological Health domain. Furthermore, the remaining three items – *forgiveness and blame, concerns about the future and death and dying*—showed higher loadings in the SRPB latent factor than in Model 1 (Table 3). Moreover, both first-order and second-order Model 2 showed a substantial improvement in fit indexes. However, results of the second-order model showed that the SRPB latent factor was the one with the lowest loading in the high second-order latent factor related to HRQoL (Table 3).

#### Internal consistency

Results of the different reliability coefficients were calculated. These showed that internal consistency was acceptable for most HRQoL dimensions (Table 4). However, the SRPB domain showed the lowest reliability coefficient. The Level of Independence domain showed an alpha below expectations (< .70). Nevertheless, the Omega coefficient (ω) showed an adequate value (.80), because it is between .70 and .90 [33]. The Omega coefficient, unlike the alpha coefficient, works with factorial loads and it makes calculations more stable [34].

**Table 4:** Reliability coefficients of the domains of the WHOQOL-HIV-BREF

Dimensions	Cronbach’s alpha	Spearman-Brown (ρ)	Guttman Split-half	MCDonald’s composite score (ω)	Intraclass correlation [IC]
Overall QoL/General Health	.77	.775	.772		.77 [.747, .794]
Physical health	.73	.662	.660	.786	.734 [.711, .756]
Psychological health	.81	.842	.827	.851	.812 [.796, .827]
Level of Independence	.67	.638	.638	.803	.678 [.650, .704]
Social relations	.75	.785	.779	.810	.749 [.728, .770]
Environmental health	.81	.809	.809	.851	.816 [.801, .830]
Spirituality/Personal beliefs	.61	.547	.545	.623	.61 [.579, .644]

#### Convergent and concurrent validity

Positive and moderate to large correlations were found between all domains and the General Health one that indicated a good convergent validity. Religion and Personal Beliefs domains (Table 3). The results of the covariances among the first-order factors are presented in Table 5.

**Table 5:** Covariances (ϕ) between the Spanish WHOQOL-HIV-BREF dimensions and correlations between these dimensions and criterion variables

	Covariances (ϕ)							Pearson’s Correlations (*r*)			
	PHY	PSY	IND	SR	EH	SRPB	GH	ART Adherence (α = .78)	Psychological Well-being (α = .91)	Negative Self-Image (α = .80)	Disclosure Concerns (α = .82)
PHY	1	.90	.96	.75	.83	.81	.67**	.44**	.63**	−.33**	−.04
PSY		1	.87	.89	.86	.95	.66**	.42**	.74**	−.44**	−.07*
IND			1	.79	.89	.74	.61**	.36**	.58**	−.31**	.00
SR				1	.90	.81	.56**	.39**	.57**	−.33**	−.07*
EH					1	.72	.65**	.47**	.57**	−.33**	−.01
SRPB						1	.41**	.29**	.53**	−.55**	−.30**
GH							1	.44**	.58**	−.29**	−.02

Notes: GH = General Health, PHY = Physical Domain, PSY = Psychological Domain, IND = Level of Independence Domain, SR = Social Relations Domain, EH = Environmental Health Domain, SRPB = Spirituality, Religion and Personal Beliefs Domain. α = Cronbach’s alpha reliability coefficient

*N* = 1462. ** *p* < .01. * *p* < .05

Regarding concurrent validity, positive correlations were found between all HRQoL dimensions and ART adherence and psychological well-being. Moreover, negative correlations were found between HRQoL dimensions and negative self-image. However, there was a moderate negative correlation with disclosure concerns when correlated with the SRPB dimension (Table 5).

#### Known-group validity

Significant differences were found according to participants’ self-reported CD4+ T cell count in several HRQoL facets and domains (Table 6). The higher the CD4+ T cell count, the higher the HRQoL scores. However, the effect sizes of the differences were small. The highest ones were found in the facets measuring *participation in leisure activities, financial resources, sexual satisfaction, symptoms of HIV,* and *overall perception of health*.

**Table 6:** Known-group comparisons of the WHOQOL-HIV-BREF scores

	CD4+ T cells					Viral load (copies mm^3^)			
	< 200 (*N* = 70)	201–400 (*N* = 153)	> 400 (*n* = 996)			Undetectable (*n* = 1321)	Detectable (*n* = 87)		Cohen’s *d*
Domains and items	Mean ± SD	Mean ± SD	Mean ± SD	*p*-Value	η^2^	Mean ± SD	Mean ± SD	*p*-Value	
Overall QoL/General Health	13.8 ± 3.8	14.1 ± 3.7	15.2 ± 3.5	.000	0.014	15.1 ± 3.5	14.2 ± 4.2	.128	0.253
How would you rate your QoL?	3.3 ± 1.2	3.4 ± 1.1	3.7 ± 1.0	.000	0.012	3.7 ± 1.1	3.5 ± 1.2	.279	0.180
How satisfied are you with your health?	3.7 ± 1.1	3.7 ± 1.0	3.9 ± 0.9	.002	0.010	3.9 ± 0.9	3.6 ± 1.1	.050	0.328
Physical health	14.7 ± 3.5	15.0 ± 3.2	15.6 ± 3.2	.004	0.008	15.6 ± 3.2	14.8 ± 3.7	.084	0.247
Pain and discomfort^a^	3.8 ± 1.3	3.9 ± 1.2	4.0 ± 1.2	.312	0.002	4.0 ± 1.2	3.8 ± 1.3	.069	0.165
Symptoms of HIV^a^	3.9 ± 1.2	4.1 ± 1.2	4.3 ± 1.1	.001	0.014	4.3 ± 1.1	4.0 ± 1.1	.078	0.272
Energy and fatigue	3.7 ± 1.1	3.8 ± 0.9	4.0 ± 1.0	.044	0.005	3.9 ± 1.0	3.9 ± 1.0	.511	0
Sleep and rest	3.3 ± 1.2	3.3 ± 1.1	3.4 ± 1.2	.190	0.002	3.4 ± 1.1	3.2 ± 1.3	.143	0.179
Psychological health	14.8 ± 3.2	14.7 ± 2.9	15.1 ± 3.0	.322	0.001	15.0 ± 3.0	14.8 ± 3.3	.633	0.066
Positive feelings	3.8 ± 1.2	3.8 ± 1.0	4.0 ± 1.0	.019	0.001	4.0 ± 1.0	3.9 ± 1.2	.575	0.009
Concentration ability	3.7 ± 0.8	3.5 ± 1.0	3.6 ± 1.0	.326	0.002	3.6 ± 1.0	3.6 ± 1.0	.708	0
Bodily image self-acceptance	4.0 ± 1.1	3.9 ± 1.0	4.0 ± 1.0	.781	0.000	4.0 ± 0.9	4.0 ± 1.0	.833	0
Self-satisfaction	3.9 ± 1.2	3.8 ± 1.0	3.9 ± 1.0	.695	0.000	3.9 ± 1.0	3.8 ± 1.1	.396	0.099
Negative feelings^a^	3.1 ± 1.1	3.4 ± 1.0	3.3 ± 1.1	.139	0.004	3.4 ± 1.1	3.2 ± 1.1	.134	0.181
Level of Independence	14.8 ± 3.5	14.7 ± 3.1	15.7 ± 3.1	.000	0.011	15.6 ± 3.1	15.3 ± 3.4	.478	0.096
Dependence on medication^a^	3.1 ± 1.5	3.1 ± 1.4	3.5 ± 1.5	.003	0.009	3.4 ± 1.5	3.3 ± 1.5	.440	0.066
Mobility	4.4 ± 0.9	4.4 ± 0.8	4.5 ± 0.8	.081	0.003	4.5 ± 0.8	4.5 ± 0.8	.969	0
Activities of daily living	3.7 ± 1.1	3.7 ± 0.9	4.0 ± 0.9	.004	0.007	3.9 ± 0.9	3.8 ± 1.0	.457	0.110
Work capacity	3.6 ± 1.3	3.6 ± 1.2	3.8 ± 1.1	.082	0.003	3.8 ± 1.1	3.7 ± 1.2	.520	0.090
Social relations	14.5 ± 3.9	14.6 ± 3.1	15.1 ± 3.1	.075		15.1 ± 3.2	14.4 ± 3.7	.081	0.216
Social inclusion	4.1 ± 1.0	4.2 ± 0.9	4.1 ± 0.9	.985	0.000	4.2 ± 0.9	4.0 ± 1.0	.161	0.220
Personal relationships	3.7 ± 1.2	3.8 ± 1.0	3.8 ± 1.0	.942	0.000	3.8 ± 1.0	3.8 ± 1.2	.822	0
Sexual satisfaction	2.8 ± 1.5	2.9 ± 1.3	3.3 ± 1.3	.000	0.013	3.3 ± 1.3	2.9 ± 1.4	.003	0.306
Social support	3.8 ± 1.3	3.8 ± 1.0	4.0 ± 1.0	.308	0.001	3.9 ± 1.0	3.7 ± 1.2	.111	0.197
Environmental health	14.7 ± 2.9	15.0 ± 2.3	15.5 ± 2.4	.005	0.003	15.4 ± 2.5	15.0 ± 2.7	.244	0.159
Physical safety and security	3.6 ± 1.1	3.7 ± 0.9	3.8 ± 0.9	.196	0.003	3.8 ± 0.9	3.6 ± 1.0	.306	0.220
Physical environment	3.8 ± 1.0	3.9 ± 0.9	4.0 ± 0.9	.279	0.002	4.0 ± 0.9	3.8 ± 1.1	.145	0.218
Financial resources	2.7 ± 1.1	3.0 ± 1.0	3.2 ± 1.1	.000	0.015	3.1 ± 1.1	3.0 ± 1.2	.241	0.090
Information for daily living	4.0 ± 1.0	4.1 ± 0.8	4.1 ± 0.8	.681	0.001	4.1 ± 0.8	4.1 ± 0.9	.754	0
Participation in leisure activities	3.5 ± 1.3	3.5 ± 1.1	3.9 ± 1.0	.000	0.019	3.8 ± 1.1	3.6 ± 1.2	.081	0.180
Home environment	3.9 ± 1.2	3.9 ± 1.0	4.0 ± 1.0	.374	0.001	4.0 ± 1.0	3.9 ± 1.1	.353	0.099
Accessibility of health services	4.4 ± 0.8	4.1 ± 0.9	4.2 ± 0.8	.111	0.003	4.2 ± 0.8	4.3 ± 0.9	.538	−0.124
Transport	3.7 ± 1.2	3.9 ± 1.0	3.9 ± 1.0	.337	0.002	3.9 ± 1.0	3.9 ± 1.0	.562	0
SRPB	14.0 ± 3.4	14.9 ± 3.3	14.5 ± 3.5	.209	0.002	14.6 ± 3.5	14.1 ± 3.7	.213	0.142
Personal life meaning	4.0 ± 1.2	4.0 ± 1.0	4.1 ± 1.0	.451	0.001	4.1 ± 1.0	4.1 ± 1.1	.926	0
Forgiveness and blame^a^	2.8 ± 1.7	3.7 ± 1.5	3.3 ± 1.6	.001	0.010	3.3 ± 1.6	3.3 ± 1.6	.974	0
Concerns about the future^a^	3.5 ± 1.3	3.5 ± 1.2	3.4 ± 1.3	.771	0.000	3.5 ± 1.2	3.2 ± 1.4	.024	0.247
Death and dying^a^	3.7 ± 1.4	3.8 ± 1.2	3.7 ± 1.2	.685	0.000	3.7 ± 1.2	3.5 ± 1.4	.283	0.164

Notes: *SRPB* Spirituality, Religion and Personal Beliefs. η^2 =^ F-test effect size. Cohen’s *d* = t-test effect size. Near to 4% (*N* = 54) of the participants stated not knowing their viral load copies, and 16.6% (*N* = 243) stated being unsure of the amount of CD4 + T cells

^a^ Reversed items recoded

Some differences according to virological status were also found. Those with undetectable viral load presented significantly higher scores in the facets measuring *satisfaction with their own health*, *pain and discomfort*, *sexual satisfaction* and *concerns about the future*, as well as marginally higher scores in facets related to *symptoms of HIV* and *participation in leisure activities*.

### Differences in HRQoL according to the characteristics of the participants

Women showed significantly lower scores than men in several HRQoL facets (see Additional file 3 for further detail). However, higher effect sizes were found in *sexual satisfaction* (Cohen’s *d* = 0.64), *bodily image self-acceptance* (Cohen’s *d* = 0.53), *participation in leisure activities* (Cohen’s *d* = 0.49), *pain and discomfort* (Cohen’s *d* = 0.44), and *energy and fatigue* (Cohen’s *d* = 0.43).

Regarding age, PLHIV older than 50 also presented lower scores in several HRQoL facets. The greater differences were found in the items measuring *sexual satisfaction* and *work capacity* (Cohen’s *d* = 0.47 and 0.37, respectively). Nevertheless, older PLHIV showed higher scores in the three HIV-specific items from the SRPB domain (*forgiveness and blame, concerns about the future, death and dying)* although the effect sizes of these differences were small (Cohen’s *d* = − 0.13, − 0.11 and − 0.10, respectively).

The scores in all facets and the statistics assessing the differences can be found in the supplementary material (Additional file 3). Differences according to the other socio-demographic characteristics revealed lower scores in heterosexuals than homosexuals in all domains (*p* < .0001) except in the SRPB domain. Bisexuals presented significant lower scores than homosexuals in General Health (*p* < .0001), Physical Health (*p* < .0001), Level of Independence (*p* < .0001), Social Relationships (*p* < .001), and Environmental Health (*p* < .0001).

Moreover, the higher the level of education, the higher the scores in all HRQoL domains (*p* < .0001) except in the SRPB domain. Besides, positive correlations were found between participants’ incomes and all dimensions of HRQoL (ranging from *r* = .11 in the SRPB domain to *r* = .36 in the Environmental Health domain; *p* < .01).

Furthermore, participants who acquired HIV infection through the injection route presented the lower scores in HRQoL domains (*p* < .0001) except in the SRPB domain.

Finally, negative correlations were found between most HRQoL dimensions (except the SRPB domain) and years since diagnosis (ranging from *r* = − 15 in the Social Relationship domain to *r* = − .27 in Physical domain; *p* < .01).

## Discussion

This study assessed the validity of the Spanish version of WHOQOL-HIV-BREF. It also described HRQoL in PLHIV living in Spain and the more vulnerable profiles.

Regarding its validity, the instrument showed acceptable construct validity although some minor changes could improve it. The SRPB domain showed the lowest reliability. In line with other studies [17, 18], the item *religion, spirituality and personal beliefs* (feeling that life is meaningful), was the one with the lowest item-domain correlation. Some authors have suggested including this item in the Psychological Health domain [16, 35]. The results of the present study support this modification.

The instrument also showed concurrent validity. In line with findings from other studies [17, 36], all of its dimensions were positively related to ART adherence and psychological well-being. At the same time, all of the HRQoL dimensions were negatively related to negative self-image, and the SRPB domain was the unique dimension that was significantly and negatively related to disclosure concerns. People who have a stigmatized condition may find some advantages in concealing the condition, but concealment may also result in mental strain and poorer psychological well-being [37, 38]. Thus, the results of this highlighted the relevance of the SRBP HIV-specific items, despite the low discriminant capability of this dimension.

The instrument was able to discriminate according to self-reported immunological and virological statuses in several HRQoL facets. This finding is in line with evidence from other studies [8, 14, 39, 40], although not all studies have had the same finding [15, 17].

The present study also described the HRQoL of PLHIV in Spain. Financial resources and sexual satisfaction were the most impaired facets. Both facets were found to be related to HRQoL in a previous Spanish study, but financial problems showed in that study the highest correlation [6]. Sociodemographic data collected from our participants revealed that they were in a precarious financial situation, and it could be damaging their HRQoL. Although Physical Health was one of the domains with higher scores, the facet related to sleep and rest had one of the lowest facet scores. This result was also found in other studies conducted in other countries [8, 14, 17, 41]. Also, HIV-specific existential concerns and negative feelings were among the most affected HRQoL facets. HIV-specific existential concerns included in the WHOQOL-HIV-BREF are related to stigma and concerns about the future and death. Research showed that there are prejudices towards PLHIV in Spain [42] and that both enacted and internalized stigma were related to poor HRQoL [6, 37]. Furthermore, research also showed that emotional loneliness, HIV-related stress and depressive mood were negatively related to HRQoL of PLHIV in Spain [6]. Stigma, depression, anxiety and other variables not measured in the present study such as comorbidities, social support, family situation or lifestyle are found to be determinants of PLHIV’s HRQoL [5]. All these variables might explain the concerns and negative feelings of the participants in the present study. Correlations found between psychological well-being, HIV-related stigma and HRQoL dimensions in this study support it.

Moreover, our results suggest that specific subgroups of PLHIV in Spain are particularly vulnerable to poor health-related quality of life for HRQoL. We found lower HRQoL scores for people who had been living with HIV for a longer time, in older people and in heterosexuals. Although incidence of heterosexual transmission of HIV has been decreasing in Spain in recent years, people whose HIV infection is attributed to this mode of transmission are estimated to constitute one-third of all PLHIV nationally [43]. Furthermore, Spain is estimated to have large proportions of PLHIV who are older than 50 years (46%) and who were diagnosed with HIV more than 15 years ago (49%) [43]. Older age and a longer period of time living with HIV are both associated with higher prevalence of non-HIV-related comorbidities such as diabetes and chronic kidney disease [44, 45]. The long-term management of multiple comorbidities, in turn, gives rise to high levels of polypharmacy [46]. Comborbidities and polypharmacy both have the potential to undermine HRQoL [47].

According to our findings, acquiring HIV through injection drug use is another factor associated with poorer HRQoL. While HIV transmission via the sharing of unsterile injection drug equipment is decreasing in Spain, 31% of PLHIV are estimated to belong to this transmission category [43]. We also found lower socioeconomic and educational status to be related to poorer HRQoL, and women in our study had lower HRQoL than their male counterparts. The role of aging should be considered in terms of how it specifically affects the HRQoL of women living with HIV, and it is notable that some of the most impaired HRQoL facets found in the present research might be exacerbated by menopause [48, 49].

Our study findings lead us to propose that initiatives to improve the HRQoL of PLHIV might have the greatest impact if they target specific populations and take into account both structural, psychosocial, and biomedical drivers of poor HRQoL. Interventions that can improve HRQoL through mechanisms such as social support and self-empowerment may have far-reaching consequences for individual PLHIV and for health systems. A recent longitudinal study found that both physical and mental HRQoL dimensions’ scores were predictive of all-cause hospitalization in a cohort of PLHIV, suggesting that improving HRQoL in this population can result in better health outcomes [50].

The main limitation of our study derives from its cross-sectional nature. Another limitation is the self-reported nature of health-related variables measured, since people may not correctly recall information such as their viral load level. The large sample size of our study led to many findings of statistically significant differences between groups. Although we reported the effect sizes, the cross-sectional nature of our study did not allow us to test how such differences have an impact on clinical outcomes. Thus, future longitudinal studies should be conducted analyzing data collected from clinical records. This would allow for assessment of the predictive validity of the instrument and thus would provide stronger evidence than the findings of the present study, as well as showing which facets and dimensions of HRQoL have the most substantial impact on clinical outcomes. Such evidence also could be used to guide interventions to address the needs of the most vulnerable populations in regard to the issues that are having the greatest negative effect on their health and HRQoL. In addition, the heterogeneity of PLHIV and the differences found point to the need to analyze scale invariances across sex, age and other relevant characteristics. Also, our study has the limitation that the population was recruited by convenience sampling. This affects the representativeness of the sample. However, the large sample size of our study could offset this limitation, as demonstrated by the finding that the characteristics of our participants were concordant with Spanish epidemiological data for PLHIV. Finally, our study did not include PLHIV having any severe psychiatric or cognitive disorder. This was because survey respondents were required to have sufficient cognitive capacity to answer the questionnaires [51]. However, there is a need to implement strategies to facilitate the participation of PLHIV who suffer from those disorders because they may be underrepresented in quality-of-life assessments, and their needs and experiences may not be taken into account in interventions to improve quality of life.

## Conclusions

This study demonstrates the validity and reliability of the Spanish version of WHOQOL-HIV-BREF. It also provides evidence about HRQoL in PLHIV in Spain by using the largest study sample to date. Long-term survivors, older adults, and women are key populations to address in order to improve HRQoL. Monitoring of HRQoL and taking steps to help patients with poor HRQoL can result in better overall health outcomes.

## Additional files

Additional file 1: Studies that have made adaptations and validations of the WHOQOL-HIV-BREF. (DOCX 33 kb)

Additional file 2: Spanish translation of the HIV-specific items of the WHOQOL-HIV-BREF. (DOCX 14 kb)

Additional file 3: Known-group comparisons of the WHOQOL-HIV-BREF scores. (DOCX 26 kb)

## Data Availability

The datasets collected and/or analyzed during the current study are available from the corresponding author upon reasonable request.

## Abbreviations

AIDS: Acquired Immune Deficiency Syndrome
ART: Antiretroviral Therapy
HIV: Human Immunodeficiency Virus
HRQoL: Health-Related Quality of Life
PLHIV: People Living with HIV
SRPB: Spirituality, Religion and Personal Beliefs domain
