# Supplementary material for: Assessing quality of life in people with HIV in Spain: psychometric testing of the Spanish version of WHOQOL-HIV-BREF
Source: Health Qual Life Outcomes. 2019 Aug 19;17:144. doi: 10.1186/s12955-019-1208-8 (PMC6700970; doi:10.1186/s12955-019-1208-8)
Supplement: Supplementary file 1 — Studies that have made adaptations and validations of the WHOQOL-HIV-BREF. (DOCX 33 kb) [file 12955_2019_1208_MOESM1_ESM.docx]

**Additional file 1** Studies that have made adaptations and validations of the WHOQOL-HIV-BREF

| Country | Authors and Year | *N* | Reliability | Internal Validity | Known-groups validity |
| --- | --- | --- | --- | --- | --- |
| Australia, Brazil, India (Bangalore and N. Delhi), Italy, Thailand, Ukraine and Zimbabwe | O’Connell & Skevington (2012) [8] | 1923 PLHIV (total)  (mean age = 33.4 years, *SD* = 9.8) | Cronbach’s α values ranged from .69 (Spiritual domain) to .82 (Environmental domain). | CFA: 6 domains (first order) and one second-order factor. | *Symptomaticity*: Five out of the six domain scores discriminated significantly among all three disease stages (asymptomatic, symptomatic and AIDS), except for the Environmental domain; the largest domain effects were for the Level of Independence and Physical domains. All facet and domain scores significantly discriminated between asymptomatic-HIV and AIDS except for “access to health and social care.” Scores decreased with disease progression. |
| China | Zhu et al. (2017)  [14] | 1100 PLHIV (mean age = 36.62 years, *SD* = 12.73)  (57 people test–retest) | Cronbach’s α ranged from .66 (Spirituality) to .85 (Level of Independence). The Cronbach’s α value of the whole scale was .93.  Test–retest reliability analysis (*n* = 57): Statistically significant ICC for all domains (*p* < 0.001), with the ICC ranging from .72 (Spirituality) to .82 (Independence). | CFA: 6 domains (first order). | *CD4+ T cell levels*: The mean scores in group CD4 count ≥ 500 cells/mm^3^ were the highest for the six domains, and the group with a CD4 count < 200 cells/mm^3^ showed the lowest scores (*p* < .05).  *Symptomaticity*: Symptomatic participants had significantly lower scores than asymptomatic participants in the Physical, Psychological and Independence domains (*p* < .05). |
| Ethiopia | Tesfaye et al. (2016) [40] | 348 PLHIV (mean age = 32.5 years, *SD* = 7.9, 25–34 years) | In the process of adaptation, new items were added while seven items were deleted because of problems with acceptability and poor psychometric properties.  The Cronbach’s α for the final tool with twenty-seven items was .93. Cronbach’s α ranged from .64 (Social relationship) to .84 (Level of Independence) after deletion of some items. | CFA: one second-order factor structure  with 6 first-order indicator factors. | *Symptomaticity*: All six domains discriminated well between symptomatic and asymptomatic people with HIV (*p* < 0.001). |
| Malaysia | Saddki et al. (2009) [35] | 157 PLHIV (mean age = 35.7 years, *SD* = 7.5, 18–67 years)  (51 people test–retest) | Cronbach’s α ranged from .70 (Social relationship) to .83 (Physical needs). The Cronbach’s alpha value of the whole scale was 0.93.  Test–retest reliability analysis (51 participants): all ICC were significant for all domains (*p* < 0.001), with ICC ranging from .60 (Social relationships) to .87  (Physical needs). | Extracting factors by principal components: 5 domains (Physical needs, Spirituality, Social relationship, Psychological, and Environmental). Their Spirituality component is comprised of 6 items (3 from the original Spirituality domain and 3 from the original Physical domain). The item “forgiveness and blame” is included in their Psychological component. | *Symptomaticity*: Asymptomatic HIV patients showed consistently higher scores than the symptomatic participants in all domains, except for Spirituality. None of the participants in the study had AIDS. |
| Portugal | Canavarro & Pereira (2012)  [16] | 1196 PLHIV (mean age = 40.73 years, *SD* = 9.69, 18–81 years) | Cronbach’s α ranged from .61 (Spirituality) to .80 (Level of Independence). | EFA: 5 domains  CFA: 6 domains (first order). | *CD4+ T cell levels*: Patients with lower CD4 count (< 200) reported significantly lower mean scores than patients with higher CD4 count (> 200), except for the Spirituality domain. Overall QoL was significantly different among three groups considered (> 200, 201–499, > 500). |
| Taiwan | Hsiung et al. (2011)  [17] | 680 PLHIV (mean age = 36.3 years, *SD* = 10.1, 17–90 years) | Cronbach’s α ranged from .67 (Physical Health and Level of Independence) to .80 (Psychological Health and Environmental Health). | CFA: 6 domains (first order) and one second-order factor. | *CD4+ T cell levels*: No significant differences were discernible between the six domain scores for those groups with different CD4 counts.  *Viral load*: Significant differences were found between the six domain scores for those groups with different viral loads (*p* < 0.05). Nevertheless, separate analyses of each domain score revealed no significant differences between the three groups.  *Symptoms’ severity*: Patients with fewer and less severe symptoms were generally found to have better QoL scores (*p* < 0.01) as well as higher domain scores in all of the WHOQOL-HIV BREF domains (*p* < .0083). |
| Thailand | Meemon et al. (2016)  [18] | 329 PLHIV (mean age = 41.95 years, *SD* = 7.82) | Cronbach’s α ranged from .45 (Level of Independence) to .80 (Environmental Health). | CFA: 6 domains (first order) and one second-order factor. | *CD4+ T cell levels*: No significant differences in any domain scores were found in patients with different CD4+ T cell levels.  *Opportunistic infections*: Patients with a history of having opportunistic infections presented a significantly higher HRQoL score in the Physical Health domain (*p* < .05) compared to those in a clinically latent stage (no history of opportunistic infections) and marginally significant Psychological Health (*p* = .054) and overall General Health Perception (*p* = .052). |
| Vietnam | Tran et al. (2012)  [39] | 155 PLHIV (mean age = 31.4, *SD* = 4.8) | Cronbach’s α ranged from  .52 (Social support) to .71 (Performance). | EFA: 6 domains (four original domains were maintained in four major parallel factors, except for ‘‘Level of Dependence’’ and ‘‘Spirituality’’; two new domains, ‘‘Morbidity’’ and ‘‘Performance’’—individual performance of functional and cognitive activities—were constructed). | - |
