# Supplementary material for: Assessing quality of life in people with HIV in Spain: psychometric testing of the Spanish version of WHOQOL-HIV-BREF
Source: Health Qual Life Outcomes. 2019 Aug 19;17:144. doi: 10.1186/s12955-019-1208-8 (PMC6700970; doi:10.1186/s12955-019-1208-8)
Supplement: Supplementary file 2 — Spanish translation of the HIV-specific items of the WHOQOL-HIV-BREF. (DOCX 14 kb) [file 12955_2019_1208_MOESM2_ESM.docx]

**Additional file 2** Spanish translation of the HIV-specific items of the WHOQOL-HIV-BREF

| English | Spanish |
| --- | --- |
| How much are you bothered by any physical problems related to your HIV infection? | ¿En qué grado te molestan los problemas físicos relacionados con tu infección por VIH? |
| To what extent are you bothered by people blaming you for your HIV status? | ¿Hasta qué punto te molesta que otras personas te culpen por tener el VIH? |
| How much do you fear the future? | ¿Cuánto temes al futuro? |
| How much do you worry about death? | ¿Cuánto te preocupas por la muerte? |
| To what extent do you feel accepted by the people you know? | ¿Hasta qué punto te sientes aceptado/a por las personas que conoces? |
