# Supplementary material for: Assessing quality of life in people with HIV in Spain: psychometric testing of the Spanish version of WHOQOL-HIV-BREF
Source: Health Qual Life Outcomes. 2019 Aug 19;17:144. doi: 10.1186/s12955-019-1208-8 (PMC6700970; doi:10.1186/s12955-019-1208-8)
Supplement: Supplementary file 3 — Known-group comparisons of the WHOQOL-HIV-BREF scores. (DOCX 26 kb) [file 12955_2019_1208_MOESM3_ESM.docx]

**Additional file 3** Known-group comparisons of the WHOQOL-HIV-BREF scores

|  | Age | |  |  | Sex |  |  |  |
| --- | --- | --- | --- | --- | --- | --- | --- | --- |
|  | < 49 years  (*n* = 912) | > 50 years  (*n* = 549) |  |  | Male  (*n* = 1159) | Female  (*n* = 282) |  |  |
| Domains and items | Mean (± SD) | Mean (± SD) | *p*-value | Cohen’s *d* | Mean (± SD) | Mean (± SD) | *p*-value | Cohen’s *d* |
| **Overall QoL/General Health** | 15.3 ± 3.6 | 14.3 ± 3.5 | <.0001 | 0.30 | 15.2 ± 3.6 | 13.9 ± 3.6 | <.0001 | 0.38 |
| How would you rate your QoL? | 3.7 ± 1.0 | 3.4 ± 1.0 | <.0001 | 0.27 | 3.7 ± 1.1 | 3.3 ± 1.0 | <.0001 | 0.37 |
| How satisfied are you with your health? | 3.9 ± .09 | 3.7 ± .09 | <.0001 | 0.25 | 3.9 ± 0.9 | 3.6 ± 1.0 | <.0001 | 0.32 |
| **Physical health** | 15.8 ± 3.2 | 14.9 ± 3.3 | <.0001 | 0.28 | 15.9 ± 3.2 | 14.2 ± 3.4 | <.0001 | 0.52 |
| Pain and discomfort^a^ | 4.1 ± 1.1 | 3.8 ± .1.2 | <.0001 | 0.23 | 4.1 ± 1.1 | 3.6 ± 1.2 | <.0001 | 0.44 |
| Symptoms of HIV^a^ | 4.3 ± 1.0 | 4.0 ± 1.1 | <.0001 | 0.26 | 4.3 ± 1.0 | 3.9 ± 1.2 | <.0001 | 0.38 |
| Energy and fatigue | 4.0 ± .97 | 3.7 ± .97 | <.0001 | 0.23 | 4.0 ± 0.9 | 3.6 ± 1.0 | <.0001 | 0.43 |
| Sleep and rest | 3.4 ± 1.1 | 3.2 ± 1.1 | .025 | 0.12 | 3.4 ± 1.1 | 3.1 ± 1.2 | <.0001 | 0.26 |
| **Psychological health** | 15.2 ± 3.0 | 14.6 ± 2.9 | <.0001 | 0.20 | 15.3 ± 3.0 | 13.9 ± 3.2 | <.0001 | 0.46 |
| Positive feelings | 4.1 ± 1.0 | 3.7 ± 1.0 | <.0001 | 0.31 | 4.0 ± 1.0 | 3.7 ± 1.1 | <.0001 | 0.29 |
| Concentration ability | 3.6 ± 1.0 | 3.5 ± .93 | .053 | 0.10 | 3.6 ± 1.0 | 3.3 ± 1.0 | <.0001 | 0.30 |
| Bodily image self-acceptance | 4.0 ± .93 | 3.8 ± .93 | <.0001 | 0.23 | 4.1 ± 0.9 | 3.6 ± 1.1 | <.0001 | 0.53 |
| Self-satisfaction | 3.9 ± 1.0 | 3.7 ± 1.0 | .019 | 0.12 | 3.9 ± 1.0 | 3.7 ± 1.1 | .001 | 0.19 |
| Negative feelings^a^ | 3.2 ± 1.0 | 3.3 ± 1.0 | .804 | -0.01 | 3.4 ± 1.0 | 3.1 ± 1.1 | <.0001 | 0.29 |
| **Level of Independence** | 15.9 ± 3.2 | 14.9 ± 3.2 | <.0001 | 0.30 | 15.8 ± 3.2 | 14.5 ± 3.4 | <.0001 | 0.40 |
| Dependence on medication^a^ | 3.5 ± 1.5 | 3.2 ± 1.4 | .003 | 0.16 | 3.5 ± 1.5 | 3.0 ± 1.5 | <.0001 | 0.33 |
| Mobility | 4.5 ± .78 | 4.3 ± .86 | .002 | 0.16 | 4.5 ± 0.8 | 4.3 ± 0.9 | .003 | 0.24 |
| Activities of daily living | 3.9 ± .97 | 3.7 ± .93 | <.0001 | 0.20 | 3.9 ± 0.9 | 3.7 ± 1.0 | <.0001 | 0.21 |
| Work capacity | 3.9 ± 1.0 | 3.5 ± 1.1 | <.0001 | 0.37 | 3.8 ± 1.1 | 3.5 ± 1.2 | <.0001 | 0.26 |
| **Social relations** | 15.3 ± 3.3 | 14.4 ± 3.2 | <.0001 | 0.29 | 15.3 ± 3.3 | 14.1 ± 3.2 | <.0001 | 0.36 |
| Social inclusion | 4.1 ± .90 | 4.0 ± .92 | .068 | 0.09 | 4.2 ± 0.9 | 4.0 ± 0.9 | .001 | 0.22 |
| Personal relationships | 3.7 ± 1.0 | 3.7 ± .99 | .435 | 0.04 | 3.8 ± 1.0 | 3.7 ± 1.0 | .359 | 0.10 |
| Sexual satisfaction | 3.4 ± 1.2 | 2.8 ± 1.3 | <.0001 | 0.47 | 3.4 ± 1.2 | 2.6 ± 1.4 | <.0001 | 0.64 |
| Social support | 3.9 ± 1.0 | 3.7 ± 1.0 | .001 | 0.18 | 3.9 ± 1.0 | 3.8 ± 1.1 | .025 | 0.09 |
| **Environmental health** | 15.5 ± 2.5 | 14.9 ± 2.4 | <.0001 | 0.24 | 15.5 ± 2.5 | 14.6 ± 2.5 | <.0001 | 0.36 |
| Physical safety and security | 3.7 ± .96 | 3.6 ± .94 | .002 | 0.16 | 3.8 ± 0.9 | 3.5 ± 1.0 | <.0001 | 0.32 |
| Physical environment | 4.0 ± .90 | 3.8 ± .89 | .003 | 0.16 | 4.0 ± 0.9 | 3.8 ± 1.0 | .021 | 0.21 |
| Financial resources | 3.1 ± 1.0 | 2.9 ± 1.0 | .001 | 0.18 | 3.2 ± 1.1 | 2.7 ± 1.0 | <.0001 | 0.46 |
| Information for daily living | 4.1 ± .85 | 4.0 ± .81 | .199 | 0.06 | 4.1 ± 0.8 | 4.0 ± 0.9 | .052 | 0.12 |
| Participation in leisure activities | 3.8 ± 1.0 | 3.5 ± 1.0 | < .0001 | 0.30 | 3.9 ± 1.0 | 3.4 ± 1.1 | <.0001 | 0.49 |
| Home environment | 4.0 ± .99 | 3.8 ± 1.0 | .006 | 0.14 | 4.0 ± 1.0 | 3.8 ± 1.1 | .002 | 0.19 |
| Accessibility of health services | 4.2 ± .79 | 4.1 ± .81 | .061 | 0.10 | 4.2 ± 0.8 | 4.2 ± 0.8 | .639 | 0 |
| Transport | 3.9 ± 1.0 | 3.8 ± .99 | .066 | 0.09 | 3.9 ± 1.0 | 3.8 ± 1.0 | .749 | 0.10 |
| **SRPB** | 14.4 ± 3.6 | 14.7 ± 3.3 | .168 | -0.07 | 14.8 ± 3.5 | 13.7 ± 3.8 | <.0001 | 0.30 |
| Personal life meaning | 4.1 ± 1.0 | 3.9 ± 1.0 | < .0001 | 0.21 | 4.1 ± 1.0 | 3.9 ± 1.1 | .015 | 0.19 |
| Forgiveness and blame^a^ | 3.2 ± 1.6 | 3.4 ± 1.5 | .010 | -0.13 | 3.4 ± 1.6 | 3.2 ± 1.7 | .057 | -0.12 |
| Concerns about the future^a^ | 3.3 ± 1.2 | 3.5 ± 1.2 | .034 | -0.11 | 3.5 ± 1.2 | 3.1 ± 1.4 | <.0001 | -0.32 |
| Death and dying^a^ | 3.6 ± 1.2 | 3.7 ± 1.1 | .062 | -0.10 | 3.8 ± 1.2 | 3.4 ± 1.4 | <.0001 | -0.32 |

Note: SRPB = Spirituality, Religion and Personal Beliefs.

^a^ Reversed items recoded
